# Draft Genome Sequence of Novel Filterable *Rhodospirillales* Bacterium Strain TMPK1, Isolated from Soil

**DOI:** 10.1128/MRA.00393-21

**Published:** 2021-07-15

**Authors:** Ryosuke Nakai, Hiroyuki Kusada, Fumihiro Sassa, Susumu Morigasaki, Hisayoshi Hayashi, Naoki Takaya, Hideyuki Tamaki

**Affiliations:** a Bioproduction Research Institute, National Institute of Advanced Industrial Science and Technology (AIST), Sapporo, Hokkaido, Japan; b Bioproduction Research Institute, National Institute of Advanced Industrial Science and Technology (AIST), Tsukuba, Ibaraki, Japan; c Department of Electronics, Graduate School of Information Science and Electrical Engineering, Kyushu University, Fukuoka, Fukuoka, Japan; d Faculty of Life and Environmental Sciences, University of Tsukuba, Tsukuba, Ibaraki, Japan; e Microbiology Research Center for Sustainability, University of Tsukuba, Tsukuba, Ibaraki, Japan; University of Arizona

## Abstract

We report the draft genome sequence of novel *Rhodospirillales* bacterium strain TMPK1, isolated from a micropore-filtered soil suspension. This strain has a genome of 4,249,070 bp, comprising 4,151 protein-coding sequences. The genome sequence data further suggest that strain TMPK1 is an alphaproteobacterium capable of carotenoid production.

## ANNOUNCEMENT

The order *Rhodospirillales* (class *Alphaproteobacteria*) is a metabolically diverse group, including acetic acid, photosynthetic purple, and magnetotactic bacteria (1, 2). Here, we report the draft genome sequence of novel *Rhodospirillales* bacterium strain TMPK1.

Strain TMPK1 was isolated from upland soil at the Tsukuba-Plant Innovation Research Center (T-PIRC), University of Tsukuba, Ibaraki, Japan. Briefly, ∼3 g soil was suspended in 27 ml UltraPure DNase/RNase-free distilled water (Thermo Fisher, Tokyo, Japan), and the suspension was filtered through a sterile 0.45-μm-pore-size filter (Millex-HV syringe filter unit; Merck Millipore, Tokyo, Japan) to target novel filterable bacteria (reviewed in reference 3). The filtered solution was spread on a gel-filled microwell array device (76 by 26 mm; pitch, 1.0 mm; well depth, 0.8 mm) composed of 900 miniature chambers for culturing microbial cells (F. Sassa, T. Kiyokawa, A. S. Utada, K. Nagata, R. Mogi, M. Hamada, T. Inaba, N. Obana, M. Yokokawa, M. Toyofuku, H. Suzuki, and N. Nomura, unpublished data) filled with 1/100-strength tryptic soy agar medium. The microcolonies formed on the device were transferred onto agar plates containing the same ingredients. Strain TMPK1 was purified by single-colony isolation.

For genome sequencing, strain TMPK1 was cultured in the R2A broth “DAIGO” (Nihon Pharmaceutical Co., Ltd., Tokyo, Japan) at 25°C for 2 weeks. DNA extraction was performed using Genomic-tip 100/G columns (Qiagen, Tokyo, Japan). A sequence library was constructed using the NEBNext Ultra II DNA library prep kit for Illumina (New England BioLabs, Tokyo, Japan) and then sequenced via 150-bp paired-end sequencing using a NovaSeq device (Illumina, Tokyo, Japan). For subsequent bioinformatic analysis, the default settings were used for all software unless otherwise specified. Raw reads (total,10,997,950 reads; ≈1.6 Gbp) were trimmed using fastp v. 0.20.0 (4) with the settings -q 5, -u 50, -n 15, and -l 150 and discarded by the in-house pipeline of Novogene Co., Ltd. (Beijing, China), according to the following criteria: reads containing (i) adapter sequences, (ii) >10% uncertain nucleotide bases, or (iii) over 50% low-quality bases (Q score ≤ 5). The remaining sequence reads were assembled using SPAdes v. 3.13.0 (5) (--careful, -k 21,33,55,77,99,127, --cov-cutoff auto). The assembled contigs were processed using the DFAST annotation pipeline v. 1.2.11 (6). The genome map with Clusters of Orthologous Groups (COGs) was analyzed and visualized using the whole-genome analysis pipeline of EzBioCloud (ChunLab, Inc., Seoul, South Korea [7]). The full-length 16S rRNA gene sequence obtained was BLASTn searched against the NCBI nucleotide/nonredundant (nt/nr) database (accessed 1 June 2021). The average nucleotide identity (ANI) of strain TMPK1 against the closest type strains identified using BLASTn was calculated using the ANI calculator (8).

**FIG 1:**
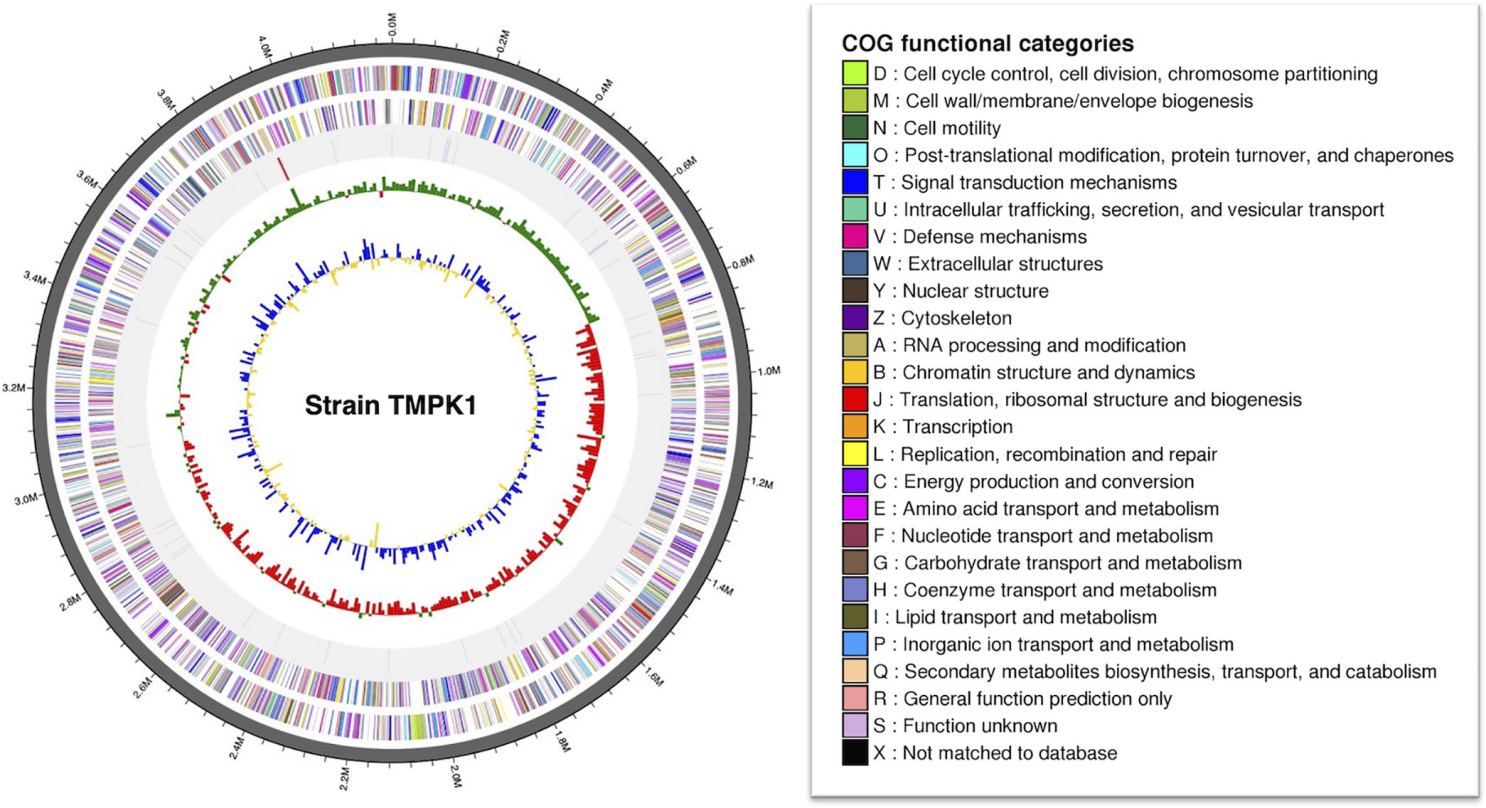
Genome map of *Rhodospirillales* bacterium strain TMPK1. The map of the longest contig (4,229,965 bp) is shown. From the circumference to the center are the annotated genes in the forward and reverse strands (the colors indicate COG functional categories [right panel]), rRNAs and tRNAs, guanine-cytosine (GC) skew metric (the mean GC-skew value is used as a baseline, with the values higher and lower than the average shown in green and red, respectively), and GC ratio metric (the mean GC ratio is used as a baseline, with the values higher and lower than the average shown in blue and yellow, respectively).

The TMPK1 genome comprises 3 contigs, 4,249,070 bp in total, 63.7% G+C content, 4,151 protein-coding sequences, 3 rRNA genes, and 49 tRNA genes, but no CRISPR. The draft genome was recovered at 381.06×. The *N*_50_ length was 4,229,965 bp. A detailed genome map is shown in Fig. 1. Strain TMPK1 is affiliated with the order *Rhodospirillales* but shares low 16S rRNA gene sequence identities (<91%) and low ANI values (<70%), with the closest type strains being Skermanella pratensis W17^T^ (9) and Haematospirillum jordaniae H5569^T^ (10) within the family *Rhodospirillaceae*. The other closest taxonomically undescribed isolate and uncultivated bacterium were identified as *Rhodospirillales* bacterium SC-11 (GenBank accession no. LC602157; 16S rRNA gene sequence identity, 99.3%) and a potassium mine soil clone (GenBank accession no. JF833841; 94.0%), respectively. These results suggest that strain TMPK1 belongs to the phylogenetically novel *Alphaproteobacteria*. Moreover, the TMPK1 genome harbors genes for carotenoid biosynthesis (e.g., phytoene desaturase gene), similar to the genomes of several *Rhodospirillales* members. The genome data presented here serve for further elucidating the phylogenetic placement and eco-physiological potentials of this strain.

### Data availability.

The TMPK1 genome sequence was deposited in the DDBJ/ENA/GenBank database under accession no. BOPV01000001.1 to BOPV01000003.1 (BioProject/BioSample no. PRJDB11251/SAMD00281908, DDBJ Sequence Read Archive [DRA] study/run no. DRP007157/DRR286848).

## References

[1] Baldani JI, Videira SS, dos Santos Teixeira KR, Reis VM, de Oliveira ALM, Schwab S, de Souza EM, Pedraza RO, Baldani VLD, Hartmann A. 2014. The family *Rhodospirillaceae*, p 533–618. *In* Rosenberg E, DeLong EF, Lory S, Stackebrandt E, Thompson F (ed), The prokaryotes: *Alphaproteobacteria* and *Betaproteobacteria*. Springer, Berlin, Germany.

[2] Hördt A, López MG, Meier-Kolthoff JP, Schleuning M, Weinhold L-M, Tindall BJ, Gronow S, Kyrpides NC, Woyke T, Göker M. 2020. Analysis of 1,000+ type-strain genomes substantially improves taxonomic classification of *Alphaproteobacteria*. Front Microbiol 11:468. doi:10.3389/fmicb.2020.00468. 32373076 PMC7179689

[3] Nakai R. 2020. Size matters: ultra-small and filterable microorganisms in the environment. Microbes Environ 35:ME20025. doi:10.1264/jsme2.ME20025. PMC7308576 32493880

[4] Chen S, Zhou Y, Chen Y, Gu J. 2018. fastp: an ultra-fast all-in-one FASTQ preprocessor. Bioinformatics 34:i884–i890. doi:10.1093/bioinformatics/bty560. 30423086 PMC6129281

[5] Bankevich A, Nurk S, Antipov D, Gurevich AA, Dvorkin M, Kulikov AS, Lesin VM, Nikolenko SI, Pham S, Prjibelski AD, Pyshkin AV, Sirotkin AV, Vyahhi N, Tesler G, Alekseyev MA, Pevzner PA. 2012. SPAdes: a new genome assembly algorithm and its applications to single-cell sequencing. J Comput Biol 19:455–477. doi:10.1089/cmb.2012.0021. 22506599 PMC3342519

[6] Tanizawa Y, Fujisawa T, Nakamura Y. 2018. DFAST: a flexible prokaryotic genome annotation pipeline for faster genome publication. Bioinformatics 34:1037–1039. doi:10.1093/bioinformatics/btx713. 29106469 PMC5860143

[7] Yoon S-H, Ha S-M, Kwon S, Lim J, Kim Y, Seo H, Chun J. 2017. Introducing EzBioCloud: a taxonomically united database of 16S rRNA gene sequences and whole-genome assemblies. Int J Syst Evol Microbiol 67:1613–1617. doi:10.1099/ijsem.0.001755. 28005526 PMC5563544

[8] Yoon S-H, Ha S, Lim J, Kwon S, Chun J. 2017. A large-scale evaluation of algorithms to calculate average nucleotide identity. Antonie Van Leeuwenhoek 110:1281–1286. doi:10.1007/s10482-017-0844-4. 28204908

[9] Guo Q, Zhou Z, Zhang L, Zhang C, Chen M, Wang B, Lin M, Wang W, Zhang W, Li X. 2020. *Skermanella pratensis* sp. nov., isolated from meadow soil, and emended description of the genus *Skermanella*. Int J Syst Evol Microbiol 70:1605–1609. doi:10.1099/ijsem.0.003944. 31904322

[10] Humrighouse BW, Emery BD, Kelly AJ, Metcalfe MG, Mbizo J, McQuiston JR. 2016. *Haematospirillum jordaniae* gen. nov., sp. nov., isolated from human blood samples. Antonie Van Leeuwenhoek 109:493–500. doi:10.1007/s10482-016-0654-0. 26857139

